# A rare case of vulvar extraskeletal myxoid chondrosarcoma: mimics and diagnostic clues

**DOI:** 10.4322/acr.2021.322

**Published:** 2021-08-20

**Authors:** Sofia S. Liou, Sanaz Memarzadeh, Sarah M. Dry, Rondell P. Graham, Neda A. Moatamed

**Affiliations:** 1 University of California Los Angeles (UCLA), David Geffen School of Medicine, Department of Pathology and Laboratory Medicine, Los Angeles, CA, USA; 2 University of California Los Angeles (UCLA), David Geffen School of Medicine, Department of Obstetrics and Gynecology, Los Angeles, CA, USA; 3 University of California Los Angeles (UCLA), Eli and Edythe Broad Center of Regenerative Medicine and Stem Cell Research, Los Angeles, CA, USA; 4 University of California Los Angeles (UCLA), Molecular Biology Institute, Los Angeles, CA, USA; 5 The VA Greater Los Angeles Healthcare System, Los Angeles, CA, USA; 6 Mayo Clinic, Department of Laboratory Medicine and Pathology, Rochester, MN, USA

**Keywords:** Chondrosarcoma, Extraskeletal Myxoid, vulva, EWSR1, NR4A3, FISH

## Abstract

Only 14 cases of extraskeletal myxoid chondrosarcoma (EMC) of the vulva have been documented in the literature. We report a case of a 63-year-old woman with EMC of the vulva confirmed by both *EWSR1* and *NR4A3* fluorescence in situ hybridization, the latter of which is a more specific probe for this entity. The unusual location of this tumor of prominent myxoid morphology gave rise to a wide differential diagnosis, which necessitated thorough histologic evaluation and confirmatory ancillary testing in the form of immunohistochemistry and cytogenetic studies. This article aims to review extraskeletal myxoid chondrosarcoma of the vulva and various diagnostic clues to help differentiate it from its histologic mimics. This is the fifth case of vulvar EMC in the literature with confirmation of a *NR4A3* gene rearrangement.

## INTRODUCTION

Malignant mesenchymal tumors of the vulva are exceedingly rare, with sarcomas accounting for only 1% to 2% of vulvar cancers.^1^ The most common sarcomas of the vulva are leiomyosarcoma, rhabdomyosarcoma, and epithelioid sarcoma. In contrast, only fourteen cases of extraskeletal myxoid chondrosarcoma (EMC) of the vulva have been reported in the literature.^2^

EMC usually arises in the proximal extremities of adult patients with a 2:1 predilection for males. The median age of onset is in the fifth decade. It is a rare, aggressive sarcoma that has a high local recurrence rate (30-50%) and metastatic potential (often pulmonary).^3^ Patients are managed with wide local excision and radiation; poor response to chemotherapy has been documented.^4^

Diagnosis of EMC is based on a combination of histopathologic features and ancillary studies. Microscopically, EMC shows a multinodular growth pattern with anastomosing cords of uniform cells surrounded by abundant myxoid matrix. Chondrocytes are notably absent, and there is accumulating evidence that EMC is unrelated to cartilage, but instead is a mesenchymal neoplasm of uncertain differentiation. Rhabdoid features, which are variably present, are associated with adverse clinical outcome. Other poor prognostic factors include old age, large tumor size (≥ 10 cm), and proximal location.^5^^,^ 6 Necrosis, hemorrhage, and hemosiderosis can be seen. The mitotic rate is usually low. Tumor cells are variably immunoreactive to S100, synaptophysin, and epithelial membrane antigen. They are negative for cytokeratins, desmin, and smooth muscle actin.^6^ CD34 has been reported to be negative in 7 cases of non-vulvar EMCs.^7^ Cytogenetically, EMC is characterized most commonly by the gene fusion *EWSR1-NR4A3*, or t(9;22)(q22;q12). This translocation can be detected by break-apart fluorescence in situ hybridization (FISH). Less commonly, the *NR4A3* gene is rearranged with *TAF15*, which appears to follow a more aggressive course.^8^

## CASE REPORT

A 63-year-old woman presented with a 3.6 cm solid mass in the right anterior perineum. The patient first noticed the lesion three years prior as a painless papule on the right vulva. The lesion persisted and gradually grew in size, now causing some pain when lying on the right side. Pre-operative magnetic resonance imaging of the pelvis showed a heterogeneously and avidly enhancing, T2 hyperintense, circumscribed mass in the right labia majora measuring 3.6 x 2.7 cm. The mass was noted to have multiple internal septations and extensive surrounding vascularity. While the mass abutted and exerted mild mass effect on the right adductor musculature, there was no frank invasion into the muscles or adjacent pelvic structures. There was no lymphadenopathy. A computed tomography-guided biopsy was performed which showed a low-grade vascular tumor with myxoid stroma, most consistent with “angiomyofibroblastoma.” Complete excision of the lesion was recommended at this time. Pelvic exam under anesthesia revealed a 4 x 3 cm mass in the right labia majora. A radical wide local excision of this mass was performed which resulted in resection of the entire lesion. The patient did well and had an uneventful postoperative course.

**Figure 1:**
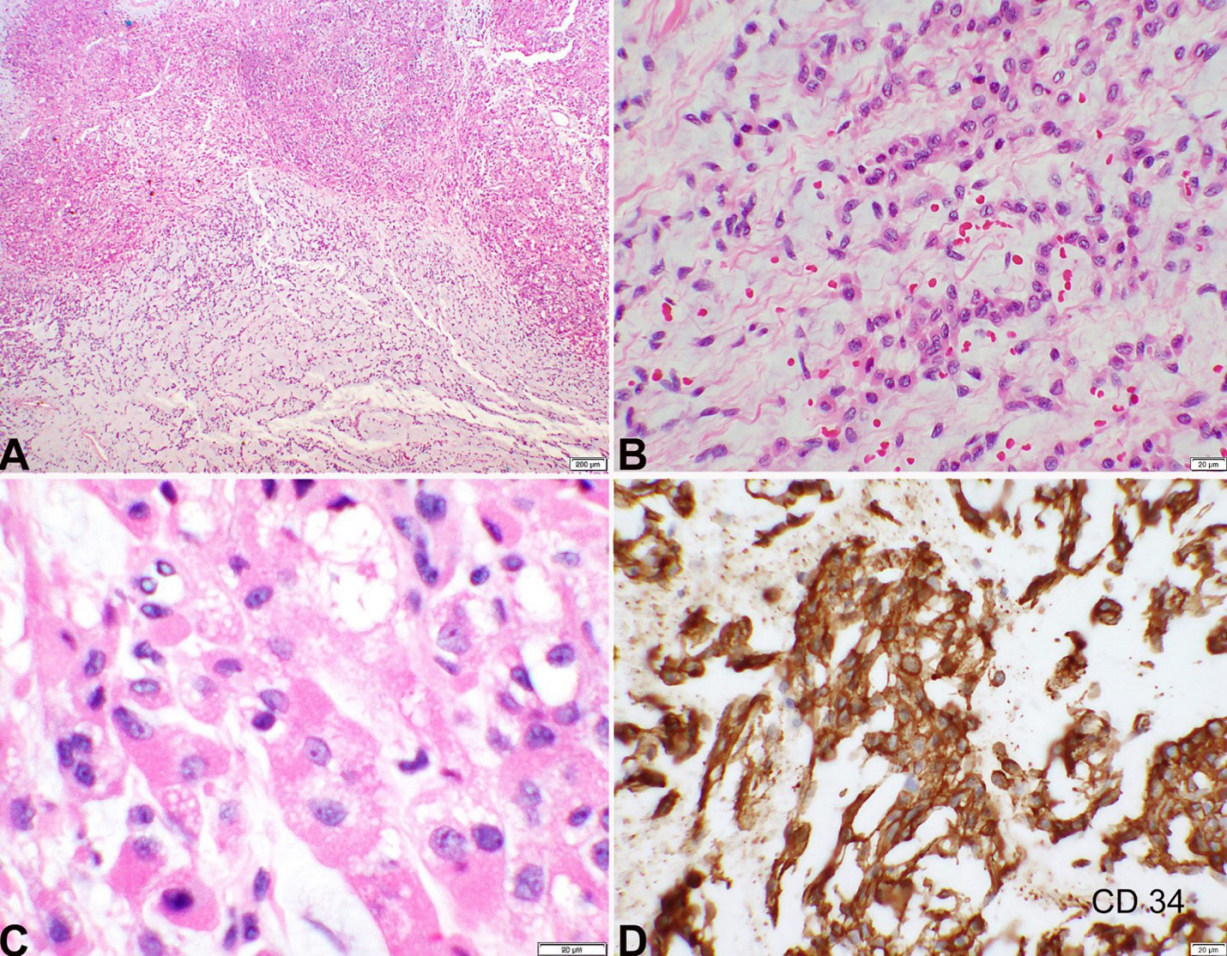
Histopathology and Immunohistochemistry. **A** – Low power photomicrograph of a hematoxylin and eosin stain shows the nodular architecture of the tumor with hypercellular and hypocellular regions with increased cellularity at the periphery of the lobules. The right side of the image shows pale blue myxoid matrix representing hypocellular areas. The left side, more eosinophilic appearing, shows both organoid and lobular growth patterns (2x objective); **B** – Abundant myxoid stroma with uniform cells arranged in trabeculae and interconnecting cords (20x objective); **C** – Rhabdoid cells with eccentrically located nuclei and eosinophilic to finely vacuolated cytoplasm (40x objective); **D** – CD34 immunohistochemical stain depicts tumor cells with intense cell membrane staining (20x objective).

Gross evaluation of the right vulvar lesion showed an irregular soft tissue fragment measuring 4.2 x 3.5 x 2.3 cm. Cut surfaces were tan-red and focally firm. Histopathology showed a spindle cell lesion with hypocellular and hypercellular areas, prominent myxoid stroma, and an overall lobulated architecture with increased cellularity at the periphery of the lobules. The cells, which were characterized by eccentrically placed round to ovoid nuclei with uniformly dispersed chromatin and eosinophilic to vacuolated cytoplasm, were arranged in clusters, trabeculae, and cords. Necrosis and mitotic activity were not seen. The tumor cells appeared low-grade and uniform without appreciable nuclear pleomorphism. The background contained abundant thin-walled vessels and extravasated red blood cells. Tumor was noted to focally infiltrate the surrounding fibrofatty tissue and was present at the inked surgical resection surface (Figures 1A-1C).

The tumor cells were strongly and diffusely positive for CD34 by immunohistochemistry (Figure 1D).

**Figure 2:**
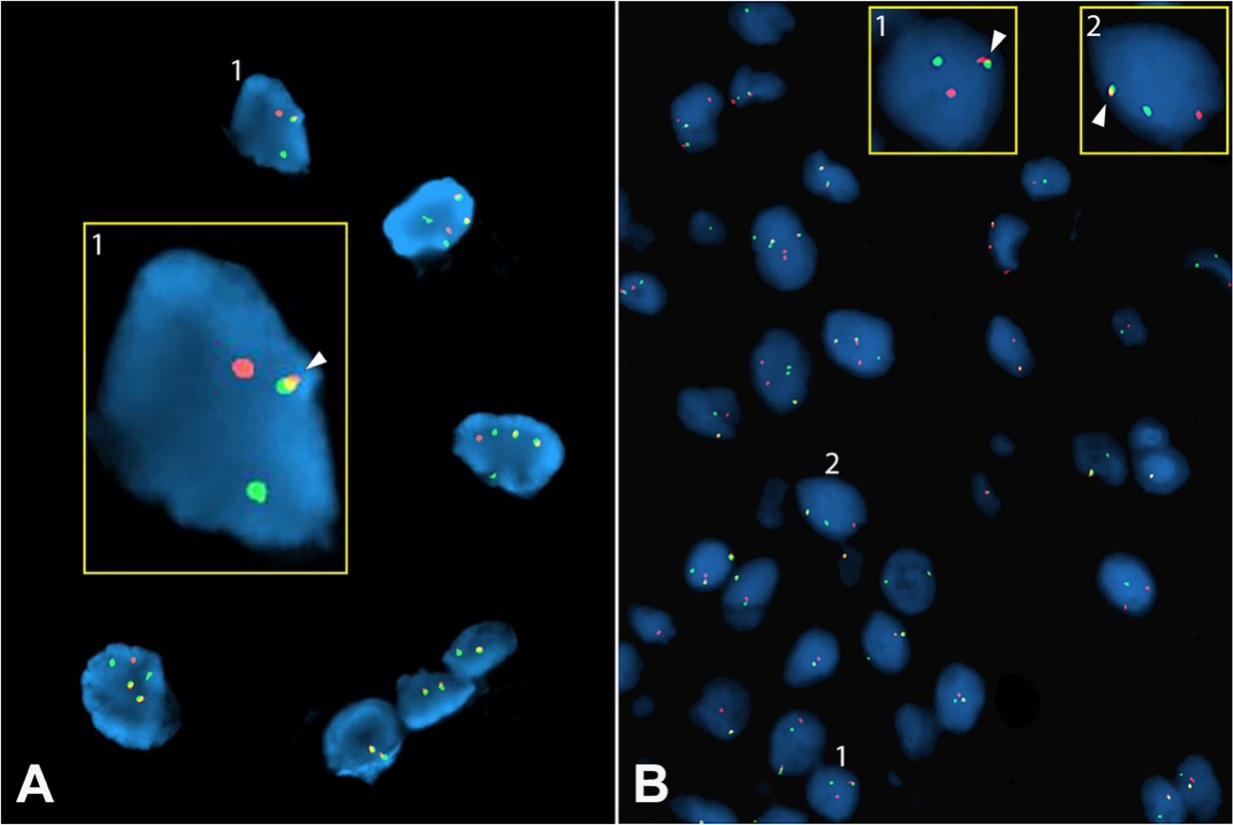
Fluorescence in Situ Hybridization (FISH). **A** – FISH analysis showing *EWSR1* gene rearrangement at the 22q12 locus. The 5’ segment of the probe is orange and the 3’ segment green. In the inset, a magnified cell nucleus from the field shows a split probe on one chromosome (two distinctly separate orange and green dots) and an intact probe (arrow) on the other (fused orange and green signals); **B** – FISH analysis depicting *NR4A3* (9q22.33-q31.1) gene rearrangement using another break-apart probe. The 5’ segment of the probe is orange and the 3’ segment is green. The arrows in the insets (1 & 2) point to the intact gene on one chromosome and a broken apart gene on the other chromosome.

Tumor cells were negative for cytokeratin, epithelial membrane antigen (EMA), smooth muscle actin (SMA), desmin, caldesmon, estrogen receptor, and S100. INI1 (SMARCB1) expression was retained. *EWSR1* FISH was performed and was positive for *EWSR1* rearrangement, consistent with a diagnosis of extraskeletal myxoid chondrosarcoma (Figure 2A). However, because *EWSR1* rearrangement can also be seen in a number of soft tissue sarcomas,^7^ confirmatory *NR4A3* FISH was performed. Positive *NR4A3* gene rearrangement by FISH was further supportive of extraskeletal myxoid chondrosarcoma (Figure 2B). Also, molecular biomarker findings at Foundation-One-Heme (Cambridge, MA, USA) included ESWR1-NR4A3 fusion and stable microsatellite status.

Post-operative imaging of the chest, abdomen, and pelvis showed post-surgical changes with no evidence of metastatic disease. The patient was referred to a sarcoma specialist with post-operative management to include a 6-week course of radiation and surveillance.

## DISCUSSION

The differential diagnosis of myxoid vulvar lesions is broad, as a significant degree of morphologic, immunophenotypic, and even molecular overlap exists among these entities. These overlapping diagnostic features make accurate diagnosis challenging, particularly on small biopsy specimens where sampling of the lesion is limited. What follows is a discussion of histologic mimics of EMC, with a focus on angiomyofibroblastoma, aggressive angiomyxoma, myxofibrosarcoma, myoepithelial carcinoma, and myoepithelial-like tumors of the vulvar region.

Angiomyofibroblastoma, a benign vulvar tumor with low rates of local recurrence, was initially favored on our biopsy specimen based on morphologic features.^9^ Angiomyofibroblastoma is characterized by alternating hypercellular and hypocellular areas admixed with small blood vessels. The stromal cells are spindled and plump, characteristically arranged around the vasculature and often are present in clusters. Lesional cells are immunoreactive to desmin and vimentin, but usually negative for cytokeratin and smooth muscle actin. While our biopsy sample had similar histopathologic and immunophenotypic features, with the addition of CD34 immunoreactivity, the excisional specimen lacked prominent aggregation of neoplastic cells around the vasculature. In addition, the histologically multinodular architecture of the excision specimen lowered angiomyofibroblastoma on the differential.^9^

Aggressive angiomyxoma is another entity that can be confused with EMC of the vulva, due to its prominent vasculature and hypocellular stroma on microscopy, as well as its much more frequent occurrence in the perineum. Aggressive angiomyxoma often presents as a large vulvar mass with a gross appearance that is ill-defined and edematous. Morphologically, the tumor consists of hypocellular stroma without atypical cells or mitotic activity, admixed with medium- to large-sized vessels with dilated lumina and occasional adventitial thickening. The tumor is often large and deep-seated with infiltrative borders, compared to its more superficial counterpart, superficial angiomyxoma, which is frequently exophytic and well-circumscribed. The cells of aggressive angiomyxoma are immunoreactive to desmin, smooth muscle actin (about half), CD34 (about half), and estrogen and progesterone receptors.^10^ While aggressive angiomyxoma entered our differential, our lesion was more cellular and had smaller vessels rather than the large- to medium-sized vessels frequently described.^10^ Furthermore, while angiomyxomas often show an edematous stroma, diffuse myxoid stroma, as seen in this case, is not typical. In addition, our lesion was immunophenotypically inconsistent given the negative staining for desmin, smooth muscle actin, and estrogen receptor.

Myxofibrosarcoma is another entity showing morphologic overlap with EMC. Myxofibrosarcoma is a more common sarcoma than EMC, and it is often found in the subcutaneous tissues of the extremities of elderly individuals. The classic morphology shows a hypocellular myxoid tumor with numerous prominent curvilinear blood vessels and lesional spindle cells that show at least focal moderate to marked pleomorphism with hyperchromatic nuclei. The lesion tends to be multinodular with infiltrative borders. Lesional cells can be immunoreactive to smooth muscle actin and CD34, but not always. They are negative for cytokeratin and desmin.^11^ In our case, the relatively uniform nuclei, architectural arrangements of cells in clusters, trabeculae and cords in addition to epithelioid and rhabdoid morphology favored EMC over myxofibrosarcoma.

Myoepithelial carcinoma is an important diagnostic consideration as well. Myoepithelial carcinomas in the soft tissue are analogous to their salivary gland counterpart. Not only do they have highly variable morphology with features overlapping with EMC, almost 50% of soft tissue myoepithelial carcinomas harbor an *EWSR1* gene rearrangement as well.^12^ Fortunately, *NR4A3* rearrangement has been negative in all cases of myoepithelial carcinoma reported in the literature to date. Thus, *NR4A3* FISH can be used to distinguish between EMC and myoepithelial carcinoma when morphologic features are inconclusive and *EWSR1* gene rearrangement is present. Immunohistochemistry can also be helpful: myoepithelial carcinomas express myoepithelial markers like p63, S100, glial fibrillary acidic protein (GFAP), and EMA, but these are not expressed in EMC.^13^

While the morphologic features of extraskeletal myxoid chondrosarcoma can conjure a wide differential, histologic clues and ancillary testing can help resolve diagnostic dilemmas. Of note, our case was strongly positive with CD34, which has not been previously reported in EMCs. Cytogenetic studies are particularly useful here, as *ESWR1-NR4A3* fusions are seen in roughly 75% of cases of EMC.^14^ *EWSR1* FISH is more widely available and thus can be useful as the initial diagnostic probe of choice. *NR4A3* has fewer fusion partners than *EWSR1* and therefore can be used as a confirmatory probe with higher specificity.^14^ So far, we have identified only 4 cases of rearranged-NR4A3 cases of vulvar EMC in the literature, making our cases the fifth report. Noguchi et al.^14^ and Flucke et al.^15^ each had two cases of vulvar EMC with confirmatory *NR4A3* rearrangement by fluorescence in situ hybridization.

## CONCLUSION

Extraskeletal myxoid chondrosarcoma of the vulva is an uncommon diagnosis in an unusual location. In addition to its rarity, the diagnosis of EMC can be challenging because of overlapping morphologic and immunophenotypic features with other myxoid mesenchymal neoplasms. Moreover, distinguishing morphologic features that are seen in excisional specimens may not be seen initially on small biopsy specimens. Representative tumor sampling thus cannot be overlooked. Cytogenetic and/or molecular studies are particularly useful for an accurate diagnosis of EMC. *EWSR1* and particularly *NR4A3* FISH analyses can help narrow this difficult differential. For the ESWR1-NR4A3 fusion, there is a targeted treatment available. Pazopanib has been reported to have anti-tumor activity in patients with progressive and advanced EMC.^16^ As our case illustrates, thorough morphologic evaluation and ancillary testing, in conjunction with imaging and clinical correlation, necessarily precede an accurate diagnosis of extraskeletal myxoid chondrosarcoma of the vulva.
